# Exposure to unhealthy product advertising: Spatial proximity analysis to schools and socio-economic inequalities in daily exposure measured using Scottish Children's individual-level GPS data

**DOI:** 10.1016/j.healthplace.2021.102535

**Published:** 2021-03

**Authors:** Jonathan R. Olsen, Chris Patterson, Fiona M. Caryl, Tony Robertson, Stephen J. Mooney, Andrew G. Rundle, Richard Mitchell, Shona Hilton

**Affiliations:** a MRC/CSO Social and Public Health Sciences, University of Glasgow, Glasgow, UK; b Faculty of Health Sciences & Sport, University of Stirling, Stirling, UK; c Department of Epidemiology, University of Washington, Seattle, WA, USA; d Mailman School of Public Health, Columbia University, New York, NY, USA

**Keywords:** Unhealthy commodity advertising, Advertising exposure, Inequalities, Transport, Spatial analysis

## Abstract

This study aimed to understand socio-spatial inequalities in the placement of unhealthy commodity advertisements at transportation stops within the Central Belt of Scotland and to measure advertisement exposure using children's individual-level mobility data. We found that children who resided within more deprived areas had greater contact with the transport network and also greater exposure to unhealthy food and drink product advertising, compared to those living in less deprived areas. Individual-level mobility data provide evidence that city- or country-wide restrictions to advertising on the transport network might be required to reduce inequalities in children's exposure to unhealthy commodity advertising.

## Highlights

• We used fixed-sized network buffers around schools and individual-level GPS data to measure exposure to unhealthy commodity advertising for children in Scotland.
• We found no evidence of socio-spatial placement for unhealthy commodity advertisements on bus stops by area deprivation or close to schools..
• Children living in more deprived areas had greater exposure to unhealthy food and drink product advertising, compared to those living in less deprived areas.
• Our evidence suggests restrictions to advertising on the transport network might be required to reduce inequalities in exposure to unhealthy commodity.
• This research is important because misunderstanding of exposure by researchers could lead to ineffective policymaking.

## 1 Introduction

Children's exposure to unhealthy commodity marketing is a global priority for policy action due to its status as a risk factor for the development of non-communicable diseases (NCDs) (World Health Organization, 2016). The literature on the commercial determinants of health identifies a range of unhealthy commodity industries, chiefly those that produce and market alcohol, tobacco and foods high in fat, salt and sugar (HFSS) (Kickbusch et al., 2016), but increasingly other health-harming industries such as gambling (Goyder et al., 2020). Advertising drives harmful consumption of alcohol (Jernigan, 2006), tobacco (Blecher, 2008) and foods high in fat, salt and sugar (HFSS) (Harris et al., 2009; Smith et al., 2019). Research suggests that children are frequently exposed to unhealthy commodity advertising (Kelly et al., 2008), and that advertisements employ techniques to which children are vulnerable (Boyland et al., 2012). Children's vulnerability to marketing communications is recognised in the United Kingdom (UK) Committees of Advertising Practice (CAP) code, which prohibits advertisers from directly encouraging children to buy any product (Advertising Standards Authority, 2007).

There is robust evidence to show that NCDs are patterned by social deprivation (Di Cesare et al., 2013) and that understanding the social patterning of exposure to unhealthy commodity advertisements may be vital to informing policies to reduce health inequalities. A wealth of international evidence demonstrates that unhealthy commodity industries target socially deprived communities (Barbeau et al., 2005; Kwate, 2007; Day and Pearce, 2011). Areas around schools and other institutions that serve children and families, such as libraries and recreation facilities, may be of relevance to health policy if they are targeted areas (Hillier et al., 2009). Evidence from Australia and New Zealand suggests that areas around schools contain disproportionately frequent advertisements for food high in HFSS (Vandevijvere et al., 2018; Kelly et al., 2008), a phenomenon that may be more pronounced around schools with higher levels of socioeconomic deprivation (D'silva, 2017; Day and Pearce, 2011). However, evidence from Scotland suggests that deprived areas may not contain disproportionate advertising for HFSS food and drink (Robertson et al., 2017). Governments have taken steps to protect under-16s from exposure to advertising of HFSS products, for example the UK Advertisements Standards Authority (ASA) guidelines ban advertising within 100m of schools (Advertising Standards Authority, 2007, 2018). Additionally, some advertisers voluntarily do not advertise within 200m of schools (Greater London Authority, 2019). Compliance with these guidelines and whether the protected areas surrounding schools should be extended has not been assessed.

Transport facilities represent a key venue of advertising, and therefore a potential target for legislation. Conventionally, mapping and monitoring aspects of the outdoor environment has relied upon physical audits performed by fieldworkers [e.g. (Sainsbury et al., 2017)]. An emerging alternative approach involves the use of publicly available datasets of panoramic photographs of outdoor locations (Bader et al., 2017). The present study involves the use of images from Google Street View as raw data for the creation of a large-scale dataset capturing advertisements on bus stops throughout the Central Belt of Scotland.

There are methodological differences in approaches to measuring exposure to unhealthy commodity advertising. The most frequently used approaches use static entities, such as administrative units or pre-defined circular, network or polygon buffers placed around fixed points, such as schools, with the aim of quantifying and describing the advertising environment there (Day and Pearce, 2011; Huang et al., 2020). These approaches have many weaknesses but there are two particularly important problems. First, living in a specific location or attending a school/work-place does not equate with exposure to *all* the environmental attributes there. Second, those who share a residential or workplace location do not necessarily experience equal exposure to the environment there; they move around. (Perchoux et al., 2013). More recently, the availability of precise location technologies, such as Global Positioning System (GPS) devices has increased and the number of studies using them to provide more accurate measures of exposure is growing. GPS data have been used to measure individual-level exposure to, for example, air pollution (Sinharay et al., 2018) and tobacco outlets (Caryl et al., 2019). This approach has not been used to explore inequalities in exposure to unhealthy commodity advertising at an individual-level. Comparing this new method with a more conventional area-level static boundary measure of exposure is important, not only to help researchers assess the utility of the new methods, but also because misunderstanding of exposure by researchers could lead to ineffective policymaking (Sadler and Gilliland, 2015).

## 2 Aims and objectives

The first aim of this study was to understand if unhealthy commodity advertisements are socially and spatially patterned, in terms of being located within pre-specified geographical distances from individuals and places, and, if such patterning was evident, whether specific types of products were more or less likely to be advertised in disadvantaged areas and near schools. The second aim was to explore individual-level socio-spatial patterning of advertisement exposure for Scottish Children aged 10–11 years old.

The specific research objectives were to:

1. Categorise the content of advertisements at bus stop locations across a large and varied geographical area.
2. Explore associations between the socio-spatial distribution of bus stop advertisements using area-based socioeconomic information.
3. Test for associations between specific categories of unhealthy commodity advertisements in the local area surrounding schools.
4. Calculate children's ‘real’ exposure to bus stop advertising using individual mobility data of Scottish children.

## 3 Methods

Our study design had three main parts:

i) To create a dataset that captured the advertising content of all bus stops within the study area.
ii) To measure the proximity of each bus stop, coded with advertising content, to schools within the same study area.
iii) To join the bus stop audit, coded with advertising content, to GPS tracks of school children, to show the adverts that children encountered as they travelled.

### 3.1 Study area and bus stop locations

The study area included the Central Belt of Scotland (1934 km^2^), incorporating the administrative boundaries of Scotland's two most populated cities, Glasgow and Edinburgh. By including two major cities and the urban/rural hinterlands between, we included a varied advertising landscape to assess that was also manageable in terms of both time and cost of auditing. The cities of Glasgow and Edinburgh contain areas which are amongst the most and least deprived areas in Scotland (Scottish Government, 2012).

Advertising at bus stops was selected because these are usually in an outdoor environment that can be virtually audited using tools such as Google Street View, as opposed to advertising within rail, subway or tram stations, which largely are indoor/covered and inaccessible for virtual audit. Bus stops also cover a wide ranging geographical area, compared to rail stations, providing a wider geographical area for our study. We extracted bus stop locations from the UK's Ordnance Survey Points of Interest dataset (code: 0732) (Ordnance Survey, 2019).

### 3.2 Bus stop audit

#### 3.2.1 Advertising rating questionnaire

A 15-item coding frame was created to categorise the main product being advertised within a visible bus stop advertisement. The key product categories to be captured by the coding frame were identified deductively based on both their prominence in literature on the commercial determinants of health (Kickbusch et al., 2016; Stuckler et al., 2012; Goyder et al., 2020) and their relevance to current policy priorities emerging from consultation with a group of representatives from health charities and public health agencies in Scotland. These categories include HFSS food and soft drinks; alcohol; nicotine products; and gambling, as well as subcategories such as confectionary and energy drinks, which were created to understand advertising of specific types of product that may be relevant to policymaking, for example potential restrictions on the sale of energy drinks to young people (Scottish Goverment, 2019).

Having established the relevant product categories deductively, the final coding frame frame/questionnaire (Supplementary Table 1) was developed inductively and iteratively based on the research team's exploratory scoping of the content of advertising on bus stops. Due to the nature of the research, rigorous, objective measurement of the nutritional content of food and non-alcoholic drink products was not possible, but sub-categories were developed that enabled the research team to be sure that close to all of the coded advertisements would contain products that would be deemed suitable for restriction in policies such as Transport for London's restrictions on the advertising of unhealthy food (Transport for London, 2019). Based on exploratory scoping of the content of advertisements, four subcategories of food and drink were deemed inappropriate for inclusion within grouped ‘unhealthy’ variables: fruit and vegetables; fruit juice or smoothie; caffeinated products; and water.

If an advertisement did not include one of the specified categories, the auditors were asked to select ‘other’, and a free text description was documented for a third of the sample of these to provide a summary of what these advertisements were. Where advertisements were illegible due to, for example, poor image quality these were selected as ‘unable to distinguish’.

#### 3.2.2 Virtual street audit

The audit was performed using the Computer Assisted Neighbourhood Visual Assessment System (CANVAS) Google Street View auditing software (https://beh.columbia.edu/street-view/). This software allows rigorous “virtual audits” that are much more rapid and cost-effective than on-location fieldwork, enabling coding of the large study area. This approach has been validated for interrater validity and concurrent validity in other contexts, such as neighbourhood audits (Bader et al., 2015; Mooney et al., 2014). Validity assessment specific to this context is described in the subsequent sub-section. A total of 10,305 bus stops were located within the study area, geocoded and imported into CANVAS. The most recent image captured by Google Street View was audited, the date of images ranged between 2008 and 2020, the majority of images audited were captured in 2019 (n:5007 (48.6% of all bus stops)), 2018 (n:2105 (20.4%)), 2016 (n:625 (6.1%)) and 2017 (n:399 (3.9%)). Three Survey Assistants were recruited as auditors and each was given a selection of randomly allocated bus stops to audit using CANVAS.

#### 3.2.3 Inter-rater reliability (IRR)

The auditors had no previous experience of using CANVAS but had previously participated in academic field-work studies, such as administering questionnaires. Auditors took part in a 1-hour face-to-face training session where they were informed of the study purpose and the advertising rating questionnaire. A series (n = 20) of images of bus stop advertisements were coded together as a group to guide them through the auditing process. Auditors then independently completed a practice audit of 60 bus stops for another UK city, Liverpool, to become accustomed to the software and rating questionnaire.

Three auditors rated the bus stops: Auditor 1: 3229 (31.3%), Auditor 2: 2748 (26.7%), and Auditor 3: 4328 (42.0%). Two hundred bus stops were audited by all three auditors, resulting in an overall percentage agreement of 92.8% and average pairwise Cohen's kappa statistic: 0.73 (95% CI: 0.68 to 0.79) (chosen for multiple raters as fixed effects (Warrens, 2010), overall figure given as individual categories were too small for valid assessment), suggesting substantial agreement between auditors (Mchugh, 2012).

### 3.3 Contextual information

#### 3.3.1 Area-level deprivation

Each bus stop was assigned the deprivation rank of the datazone within which it was located. Datazones are small areal units used in the production of official statistics in Scotland. They contain populations of between 500 and 1000 household residents (Scottish Goverment, 2006)). Deprivation rank was assigned using the Income Domain of the 2016 Scottish Index of Multiple Deprivation (SIMD) (Scottish Government, 2012). The Income Domain measures low income as indicated by the receipt of government benefits and was chosen over the full SIMD as that includes an element of geographical and facility accessibility which may have biased our results. The datazone income ranks were grouped using a binary deprivation variable (least deprived/most deprived) in which the three most deprived quintiles were grouped into the least deprived category for the Central Belt of Scotland area.

#### 3.3.2 Distance to schools

The locations of all schools within the Central Belt of Scotland were extracted from Ordnance Survey points of interest classification (code: 2031). Each school location was plotted within ArcMap 10.6. The Scottish Road and Path network was obtained from the Ordnance Survey MasterMap Integrated Transport Network Layer. Using the Network Analyst extension with ArcMap 10.6, 100-meter (m), 200m, 800m road and path network buffers were created surrounding each school location, and a dichotomised (yes/no) variable was created for each bus stop to identify whether it was located within any of the distance buffers. The 100m network buffer was selected based on the UK Advertisements Standards Authority (ASA) guidelines to protect under-16s from exposure to advertising of HFSS products within 100m of schools (Advertising Standards Authority, 2007, 2018). A 200m buffer was chosen due to some advertisers (for example, McDonalds) voluntarily not advertising within 200m of schools (Greater London Authority, 2019). A larger 800m buffer was used to assess advertising across a wider geographical area, as the Scottish Government have requested that the Advertising Standard Authority (ASA) prohibit advertising of HFSS products within 800m of locations frequently accessed by children (such as schools and leisure centres) (Scottish Goverment, 2018).

### 3.4 Children's individual-level mobility data

#### 3.4.1 SPACES

We used data from participants in the ‘Studying Physical Activity in Children's Environments across Scotland’ (SPACES) study (Mccrorie et al., 2017) who were recruited from the Growing Up in Scotland (GUS) study—a nationally representative longitudinal cohort study originating in 2005. From a possible 2402 children who participated in GUS sweep 8 interviews, 2162 consented to be approached by SPACES researchers, of which 51% (n = 1096) consented to take part. Participants were provided with an accelerometer (ActiGraph GT3X+) and a GPS (QstarzSTARZ BT-Q1000XT; Qstarz International, Taiwan) and asked to wear them over eight consecutive days between May 2015 and May 2016 when the participants were 10–11 years old. SPACES inclusion criteria required at least four weekdays of accelerometer data and one day of weekend data, resulting in a subset of 774 participants. Of these, 229 participants (54% female) resided in the Central Belt study area and met our inclusion criteria of providing at least 1 h of GPS data.

#### 3.4.2 Area-level deprivation

A measure of area-level socioeconomic deprivation for the datazone containing each participant's home address was assigned to each child using the Income Domain of the SIMD. Due to under-representation of children from the most deprived areas we created a binary deprivation variable (least deprived/most deprived) in which the three most income deprived quintiles were grouped into the least deprived category (30% of participants).

### 3.5 Statistical analysis

#### 3.5.1 Bus stop advertisement category

The number and proportion of all advertisements by category were described for all 15 main categories (Table 1). Due to some categories having a small number of advertisements and to provide relevant outcomes for policy makers, aggregation was conducted. The following categories were use in the subsequent analyses:

- Unhealthy food and/or drink, including sugar-sweetened beverages, fast food, confectionary, crisps and savoury snacks, cakes, pastries, puddings and sweet biscuits, and ice-cream/frozen desserts.
- Unhealthy food, including fast food, confectionary, crisps and savoury snacks, cakes, pastries, puddings and sweet biscuits, and ice-cream/frozen desserts.
- Sugar-sweetened beverages.
- Alcohol product.
- E-cigarette product.
- Gambling.

**Table 1:** Summary of bus stop advertisement categories.

Advert	Number	Percent
Other	1764	56.5
FOOD Fast food product	478	15.3
FOOD Confectionary	211	6.8
DRINK Alcohol	124	4.0
DRINK Sugar-sweetened beverage	120	3.8
DRINK Water	103	3.3
FOOD Ice cream and frozen desserts	71	2.3
DRINK Fruit juice or smoothie	52	1.7
FOOD Crisps and Savoury snacks	34	1.1
DRINK Caffeinated products	33	1.1
FOOD Cakes or pastries or puddings or sweet biscuits	31	1.0
Unable to distinguish	31	1.0
DRINK Artificially sweetened beverage	27	0.9
E-cigarettes	22	0.7
Gambling	14	0.4
FOOD Fruit and vegetables	5	0.2
DRINK Energy drinks	3	0.1
*Total*	*3123*	*100*

Free-text descriptions of 467 (of 1764) advertisements classified as ‘other’ were also summarised into overall categories and supplied as Supplementary Table 2.

The number of advertisements by the area-level deprivation (least deprived/most deprived) were described and a Chi-Square test performed to test whether there was a relationship between these variables. Logistic regression was performed to explore whether advertisements were more or less likely to be located within areas based on deprivation, each advertising category was modelled individually.

#### 3.5.2 Proximity to schools

A binary logistic regression model was used to predict the odds of a bus stop advertisement containing a specific product category, for example unhealthy food, within a 100m, 200m of 800m buffer of all schools. Individual product categories were modelled discretely.

#### 3.5.3 Individual level exposure of children to bus stop advertisements

The straight-line distance from each GPS location to every bus stop location was measured using the *sf* package in R (Pebesma, 2018). The nearest bus stop to each GPS location was retained along with information about the advertisements at that stop. Using a novel methodology (Caryl et al., 2019), GPS locations were classed as ‘exposed’ when distance to nearest bus stop containing an advertisement was <10m. The 10m threshold was used because this is the distance a child walking at 1 m/s (3.6 kph) would travel between each GPS location. Participants were asked to wear GPS devices during waking hours, leading to variation in daily wear time. To account for this, we standardised rates of exposure by modelling counts of exposed GPS locations for each participant with total wear time (e.g. total GPS locations) as an offset. Exposure rates of each participant to each category of advertisement were compared between the binary income deprivation levels with negative binomial generalised linear models to account for overdispersion.

To determine which response variables would have a sufficient sample size to model, an alpha = 0.05 with a 50% probability (of failing to detect a difference of a small effect (0.5 x standard deviation)) required 16 individuals per group to be exposed to specific advertising categories (Croarkin et al., 2006), therefore individual-level exposure was not performed for gambling and e-cigarette advertisements.

Comparison of our sample with the national level demographic distributions indicate slight under-representation of children from the two most deprived quintiles and over-representation of the least deprived quintiles. However, after applying individual-level cross-sectional weights that were generated for all GUS respondents in sweep 8 (Mccrorie et al., 2017), our sample could be considered nationally representative.

In addition to socioeconomic status, we also included control variables for children, sex; the season in which they were tracked (winter: October–March); and whether their residence was in an urban or rural setting, following Mccrorie et al. (2020). For the latter, we used the Scottish Government's six-category classification system, which considers both population size of the settlement and remoteness/accessibility (based on drive time to the nearest settlement with a population of 10,000 people or more) (Scottish Goverment, 2006). Settlements are defined as a group of high-density postcodes (i.e. more than 2.1 residential addresses per hectare, or population per hectare greater than five) whose combined population rounds to 500 people or more (National Records Of Scotland, 2016). They are separated by low density postcodes. To ensure sufficient sample size within groups, we dichotomised the six-category classification system into two categories (urban, rural), each comprising three of the original classes.

Models were fully adjusted for income deprivation, urbanity, sex and season. Results are presented as exponentiated coefficients (transformed back to response scale as models were negative binomial using a log link function) and effects are presented for income deprivation, urbanity, sex, and season (Reference categories: Income deprivation = least deprived; Urbanicity = urban; Sex = male; Season = winter) by advertisement category. All Individual-level exposure analyses were performed in R-4.0.0.

## 4 Results

### 4.1 Bus stops

All 10,305 bus stop locations were audited, of which 9701 (94.1%) actually contained a visible bus stop. Of the 9701 bus stops, 7856 (80.9%) did not contain an advertisement, 532 (5.3%) had one visible advertisement, 1294 (13.7%) two advertisements, and 19 (0.2%) had three advertisements. A total of 1845 bus stops had one or more advertisements and all of these were subsequently categorised.

### 4.2 Advertisement categories

1845 bus stops were audited, totalling 3123 advertisements as some bus stop locations had one or more advertisements. Fast food products totalled 15.3% of all advertisements (n = 478), confectionary 6.8% (n = 211) and alcohol 4.0% (n = 124) (full list of advertisements: Table 1). Over half of the advertisements (n = 1,764, 56.5%) were ‘other’ and 427 of these were described using free-text and are summarised in Supplementary Table 2.

### 4.3 Socio-economic patterning of advertisements

Table 2 presents the grouped advertisement categories by area level socio-economic deprivation, highlighting that, in terms of advertisement location and type of advertisement, there did not appear to be a social relationship. A similar pattern was displayed in the results of the logistic regression models (Supplementary Table 3).

**Table 2:** Advertisement category by area level socio-economic deprivation.

Advertisement	Area-level income deprivation				Chi2	p value
	Most deprived		Least deprived			
	n	%	n	%		
Unhealthy food and/or drink beverages	574	60.7	371	39.3	1.72	0.19
Unhealthy food	500	60.6	325	39.4	1.66	0.20
Sugar-sweetened beverages	*74*	*61.7*	*46*	*38.3*	*0.04*	0.85
Alcohol	78	62.9	46	37.1	0.01	0.92
E-cigarettes	13	59.1	9	40.9	0.11	0.74
Gambling	10	71.4	4	28.6	0.48	0.49
Other	*1121*	*63.6*	*643*	*36.5*	*1.80*	0.18
All (with adverts)	2084	62.5	1251	37.5	–	***-***

### 4.4 Advertisement proximity to schools

The likelihoods of each advertisement category being displayed within a 100m, 200m and 800m network buffer around schools are presented in Table 3. The results indicate that it is very unlikely that unhealthy products were advertised within the school environment. This was consistent from a 100m–800m network distance of schools. A similar pattern was found for e-cigarette advertising, where there were no advertisements within 100m or 200m, and very unlikely to be within 800m of a school (OR:0.33, 95% CI 0.13 to 0.86). However, there was an increased likelihood of gambling advertising within 100m of schools. There was an increase of ‘other’ products being advertised around schools (100m: OR:3.20, 95% CI 2.00 to 5.11), as we may expect when ‘unhealthy’ products are less likely to be advertised here; the advertising space must be used.

**Table 3:** Likelihood of advertisement within 100m, 200m and 800m network buffer around schools.

Advertisement	School within 100m			School within 200m			School within 800m		
	OR	95% CI	p value	OR	95% CI	p value	OR	95% CI	p value
Unhealthy food and/or drink beverages	*0.15*	*0.07 to 0.35*	*<0.001*	*0.32*	*0.27 to 0.46*	*<0.001*	0.87	0.69 to 1.10	0.236
Unhealthy food	***0.15***	***0.06 to 0.37***	***<0.001***	***0.32***	***0.24 to 0.44***	***<0.001***	0.96	0.75 to 1.24	0.777
Sugar-sweetened beverages	0.26	0.04 to 1.85	0.178	0.74	0.41 to 1.33	0.314	***0.58***	***0.36 to 0.95***	***0.029***
Alcohol	0.25	0.03 to 1.79	0.167	***0.25***	***0.10 to 0.62***	***0.003***	1.20	0.65 to 2.18	0.574
E-cigarettes	–	–	–	–	–	–	***0.33***	***0.13 to 0.86***	***0.023***
Gambling	***5.31***	***1.17 to 24.05***	***0.030***	2.47	0.77 to 7.93	0.127	–	–	–
Other	***3.20***	***2.00 to 5.11***	***<0.001***	***2.7***	***2.18 to 3.36***	***<0.001***	***1.23***	***0.99 to 1.53***	***0.1***

### 4.5 Children's exposure to advertisements and advertising types by socio-economic deprivation

**Fig. 1:**
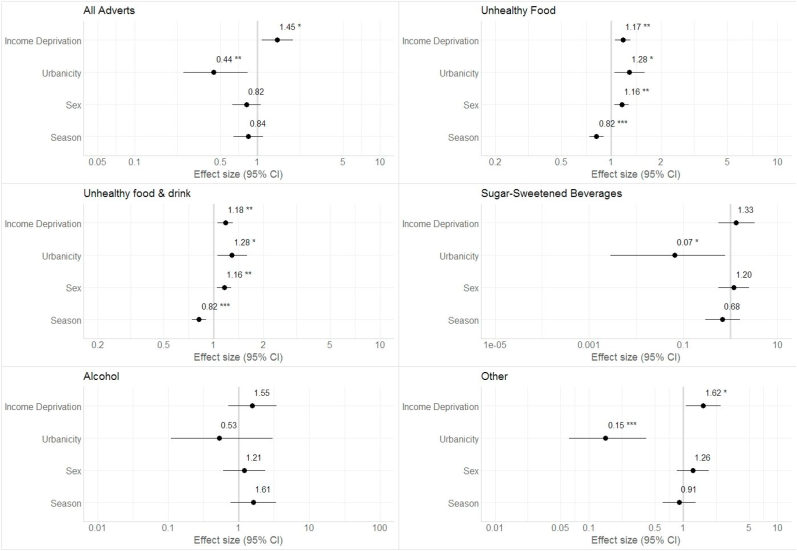
Effect size (i.e. mean difference) and 95% CIs between exposure to advertisement categories for children by area-level income deprivation, urbanity, sex, and season (Reference categories: Income deprivation = least deprived; Urbanicity = urban; Sex = male; Season = winter). Note: Models are fully adjusted for deprivation, urban, sex and season. Where 95% CI for coefficients intercepts one, there is no difference in exposure between income deprivation levels, urbanity, sex, and season. Where the 95% CIs fall above one, it indicates that children in, for example, the most deprived areas experienced greater exposure, compared to the least deprived. Statistical significance: ** p < 0.01; * p < 0.05.

Full outputs from models comparing exposure to advertisement categories across binary income deprivation levels, urbanicity, sex and season are shown in Supplementary Table 4, while summary outputs are shown in Fig. 1. These indicate that children living in the most deprived areas encountered bus stops (with or without advertisements) significantly more frequently than those in the least deprived areas (coefficient (coef): 1.45, 95% CI 1.09 to 1.95). They also experienced significantly greater exposure to unhealthy foods (coef: 1.18, 95% CI 1.06 to 1.31), unhealthy foods and drink (coef: 1.18, 95% CI 1.0 to 1.31), and ‘other’ advertisements (coef: 1.62, 95% CI 1.07 to 2.46).. Children living in rural areas were exposed to less advertisements regardless of type (coef: 0.44, 95% CI 0.24 to 0.81) than children residing in urban areas. Children living in urban areas had greater exposure to unhealthy food (coef: 1.29, 95% CI 1.04 to 1.31) and unhealthy food and drink (coef: 1.29, 1.04 to 1.31) advertising than those living in rural areas. Fig. 1 also indicates that while some models did not reach statistical significance (due to small sample sizes) there was evidence of more socioeconomic patterning (Sullivan and Feinn, 2012), for example, Alcohol and Sugar-sweetened beverage advertising for children from the most deprived areas.

## 5 Discussion

The primary aim of this study was to understand whether there is socio-spatial inequality in the distribution of unhealthy commodity advertisements. In terms of advertisement location and type of advertisement, we did not find an association with the area based deprivation measures. We also found that unhealthy commodity advertisements were unlikely to be located around schools, which was consistent from a 100m–800m network distance surrounding schools. Together, these results indicate no bias towards more deprived areas or schools in the locations of unhealthy commodity advertisements on bus stops in the Central Belt of Scotland.

Our secondary aim was to measure ‘real’ exposure to unhealthy commodity advertisements using individual mobility data of children aged 10/11 years old. Here, we found that children who resided within more deprived areas had greater contact with the transport network and also evidence for socio-economic inequalities in exposure to advertisements. Children from more deprived areas were more likely to be exposed to unhealthy food and unhealthy food and drink product advertising compared to those living in less deprived areas.

A recent study in New Zealand, using an area-based design, assessed unhealthy commodity advertisements for transport stops within a 500m walking distance of schools and found there to be no association between unhealthy commodity advertisements around schools or a socio-spatial patterning, the results were opposite to the authors’ hypothesis, finding that advertising increased as distance from school also increased (Huang et al., 2020). In Austria, a 950m walking buffer surrounding schools was applied and found child-oriented snacks were not more frequently advertised there (Missbach et al., 2017). A recent systematic review found adherence to voluntary codes of practice for online and on television advertising prior to 2013 to be high in the UK but this was not universal in all countries (Galbraith-Emami and Lobstein, 2013) and a North American study showed city-level variation in area specific advertisements (Hillier et al., 2009). Given the global nature of the unhealthy commodity advertising and NCD crisis, future research using the methodology applied here for cities in a range of countries could further strengthen the body of evidence of unhealthy commodity advertising exposure.

We found that children from more deprived areas had greater exposure to bus stops and the transport network. In combination with our observation of no association between school proximity and unhealthy commodity advertisement, this suggests that transport network wide restrictions of unhealthy commodity advertisements, rather than school-based spatial restrictions, may be effective to target inequalities in exposure for children. A previous study has found that transportation stops and roads were an important component of where 10-11-year-old children living in Scotland spent their time (Olsen et al., 2019). However, that study did not explore differences by socioeconomic status, so this analysis provides important additional insights. Further evidence for socioeconomic inequalities in exposure to unhealthy commodity advertising also stems from Scotland (Robertson et al., 2017) but also globally, for example in Sweden (Fagerberg et al., 2019), Australia (Settle et al., 2014), North America (Lowery and Sloane, 2014) and the UK (Thomas et al., 2019). Scottish data for 2017 show that a higher proportion of children from more deprived areas used a service bus to travel to school than those from less deprived areas (most deprived quintile: 9%; least deprived quintile: 5%), a similar but larger difference was found by net household income (up to £15,000: 12.2%; over £40,000: 2.9%) (Scottish Government, 2018). As well as socioeconomic inequalities, racial inequalities in advertising outside schools in North America have been highlighted where Hispanic schools had significantly more food and beverage advertisements outside than other schools (Herrera and Pasch, 2018).

Marketing targeted at children has been labelled an unfair exploitation of children's inherent vulnerabilities (Kunkel et al., 2004). Children's cognitive development limits their ability to differentiate between marketing messages and reality (Ludvigsen and Scott, 2009), and makes them particularly susceptible to persuasion from advertising (Rozendaal et al., 2010). Further, evidence suggests that unhealthy commodity advertising has cumulative effects on children, with attitudes, choices and consumption behaviours correlating with frequency of exposure to marketing messages (Scully et al., 2012; Gordon et al., 2011). Given children's heightened vulnerability to marketing messages, it has been suggested that unhealthy commodity advertising which targets children breaches their rights to appropriate information, protected by the United Nations Convention for the Rights of the Child (Smith et al., 2019; United Nations, 1989). From this perspective, restricting unhealthy commodity advertising in settings frequented by children would represent a priority for public policy. Transportation networks represent a key component of the built environment within which marketing could be restricted, particularly when the cumulative effects of children's exposure to promotion of unhealthy commodities are considered.

Since 2019, Transport for London (TfL) has prohibited advertising for HFSS foods on bus stops, taxis and aspects of their transport network, covering 32 London boroughs and representing 30 million daily journeys (Transport for London, 2018). Advertising for alcohol is prohibited on public transport, bus stops and stations in Ireland (Alcohol Action Ireland, 2019), and on bus shelters and other local authority property in New York City (City Of New York, 2019). Based on our findings, we would recommend similar city-level or national policy restrictions were implemented on transport networks in Scotland and elsewhere.

Our findings hold significant policy importance. They highlight that when measuring inequality in exposure to unhealthy commodity advertisements, the sole use of area-level measures of socio-economic situation may be insufficient.

## 6 Strengths and limitations

Our study has a number of strengths. We conducted a virtual audit covering a substantial (1934 km^2^) geographical area containing 10,305 bus stops. We were able to conduct a novel analysis using individual-level mobility data of children collected using precise GPS devices which could be linked to our advertising audit. This allowed us to compare area- or individual-based measures of exposure for unhealthy commodity advertising, a key strength of this study design. The methods used here can be applied elsewhere to provide evidence of the spatial nature of unhealthy commodity advertising in different contexts and provide evidence of relevance to policy.

We noted several limitations to our study. We did not conduct a nutrient profiling model or similar to categorise healthy/unhealthy – just visual identification of a products' likely categorisation. We mitigated this by keeping more controversial categories (yoghurts, coffee) out of the combined ‘unhealthy foods’ category as in other studies (Huang et al., 2020; Sainsbury et al., 2017).

As we collected data using Google Street View auditing software, we were only able to collect information about advertising at bus stops, we omitted other outdoor advertising (such as: billboards, monoliths, buses, taxis, repurposed phone boxes), broadcast advertising, print advertising and the rapidly-growing world of online advertising/marketing. Google Street View data have temporal variation between images; however, this introduces a randomness to the sample that may smooth out temporary anomalies and seasonal variations in advertising practices. However, this should also be noted as a limitation of the study design as the outcome may exhibit spatial autocorrelation due to not being able to control what season street imagery was captured, which will likely display a spatial pattern as geographical areas may be audited on the same day. The images audited from Google Street View were captured across a significant time period, the majority of the images audited here were captured between 2017 and 2020 (73.1% of sample), and the earliest image during 2008. This can be viewed as both a strength and limitation as it creates random exposure measurement error and bias to the null, therefore the exposure may be larger than our results suggest. The individual-level data of children's movements were collected during 2015 and 2016, prior to the collection of the majority of the advertising images. As the children are aged 10–11 years and nationally representative cohort, it is unlikely for this age group mobility patterns have changed significantly.

## 7 Study context in relation to the outbreak of 2019 novel coronavirus disease (COVID-19)

The global COVID-19 pandemic may have two outcomes relevant to this research. Firstly, increased public discourse around resource limitations within national health providers/services may increase political and public acceptance of policy approaches to the prevention of obesity and other NCDs. Emerging evidence suggests that obesity and other underlying NCDs worsen the acuteness of COVID-19 (Simonnet et al., 2020; Ryan et al., 2020; Dietz and Santos-Burgoa, 2020). Further, the UK Prime Minister, who had previously sought to reframe policies such as the Soft Drinks Industry Levy as ‘sin taxes’ (Iacobucci, 2019), reportedly now favours policy interventions to tackle obesity (Tunnidge, 2020). Restrictions on movement of people enforced by governments worldwide has led to less people on public transport and in public settings (Google LLC, 2020), therefore exposure, at least temporarily, will likely have been reduced. However, this may increase advertising exposure in other settings for children, such as online, in print and television which is worthy of investigation.

## 8 Conclusions

We found no evidence for placement of unhealthy commodity advertisements in more deprived areas or around schools in the Central Belt of Scotland. This suggests that school-based restriction boundaries alone, in addition to current ASA restrictions that have likely been effective in reducing advertising in these locations, would be ineffective in reducing children's exposure to unhealthy commodity advertising. However, our novel application of individual-level mobility data provides evidence that city- or country-wide restrictions to advertising on the transport network might be required to reduce inequalities in exposure to unhealthy commodity advertising for children. This research, using different advertising exposure measures, is important because misunderstanding of exposure by researchers could lead to ineffective policymaking.

## Funding statement

JRO, FC & RM are part of the Places and Health Programme (MC_UU_00022/4; SPHSU10), CP & SH are part of the Policy Programme (MC_UU_12017/15; SPHSU15), both at the MRC/CSO Social and Health Sciences Unit (SPHSU), 10.13039/501100000853University of Glasgow, supported by the 10.13039/501100000265Medical Research Council and the Chief Scientist Office (Scotland). The CANVAS tool is supported by a grant from the Eunice Kennedy Shriver National Institute of Child Health and Human Development (P2CHD058486), awarded to the 10.13039/100011967Columbia Population Research Center.

## Declaration of competing interest

The authors declare that there are no conflicts of interest.
