# Supplementary material for: Exposure to unhealthy product advertising: Spatial proximity analysis to schools and socio-economic inequalities in daily exposure measured using Scottish Children's individual-level GPS data
Source: Health Place. 2021 Mar;68:102535. doi: 10.1016/j.healthplace.2021.102535 (PMC9227708; doi:10.1016/j.healthplace.2021.102535)
Supplement: Multimedia component 1 [file mmc1.docx]

**Supplementary Materials.**

**Supplementary Table 1: Advertising rating questionnaire – 15-item Coding frame**

**CANVAS Questionnaire version 1**

Q1. Do you see a bus stop?

- Yes
- No (If no, ends here)

Q2. How many bus stop adverts do you see?

- 0 (If 0, ends here)
- 1
- 2
- 3

Advert #1

Q3. What does advert 1 contain?

- No Advert
- Unable to distinguish (Flags for additional check)
- FOOD Fast food product (For example McDonalds or KFC or subway)
- FOOD: Confectionary (for example ‘Dairy Milk’ or ‘Fruit Pastilles)
- FOOD: Crisps and savoury snacks.
- FOOD: Cakes or pastries or puddings or sweet biscuits.
- FOOD: Fruit and vegetables
- FOOD: Ice-cream and frozen dessert
- DRINK: Sugar-sweetened beverage (for example ‘Coca-Cola’ or ‘Irn Bru’)
- DRINK: Artificially sweetened beverage (for example ‘Diet Coke’ or ‘Pepsi Max’)
- DRINK: Alcohol (Wine, beer, spirits)
- DRINK Energy drinks (for example Monster or Red Bull)
- DRINK: Fruit juice or smoothie
- DRINK: Caffeinated products (for example ‘Starbucks’ or Costa Coffee’)
- DRINK: Water (including flavoured water)
- Gambling (for example ‘National Lottery’ or ‘Paddy Power’ or ‘Gala Bingo’)
- E-cigarettes (or E-cig liquid)
- Other

……………………………………….

Q4. Does Advert 1 contain a fast food brand?

- Yes
- No

Q1. Does Advert 1 contain a supermarket or food store?

- Yes
- No

**Supplementary Table 2: Free text advertisement category responses for of adverts categorised as ‘other’ (427 (a third classed as other) were categorised using free text)**

| **Advertisement category** | **Number** | **Percent** |
| --- | --- | --- |
| Finance | 51 | 11.9 |
| Phone, Broadband, tv. | 46 | 10.8 |
| Tv, Film or cinema | 43 | 10.1 |
| Travel | 39 | 9.1 |
| Health and Beauty | 38 | 8.9 |
| Supermarket | 38 | 8.9 |
| Yoghurt | 32 | 7.5 |
| Charity | 30 | 7.0 |
| Clothing | 20 | 4.7 |
| Cold tea drink | 14 | 3.3 |
| DIY | 13 | 3.0 |
| Social network | 7 | 1.6 |
| Fast-food - non-food | 6 | 1.4 |
| Takeaway provider | 6 | 1.4 |
| Electronics | 5 | 1.2 |
| Mayo or sauce | 5 | 1.2 |

**Supplementary Table 3: Likelihood of unhealthy commodity advertisement by area level socio-economic deprivation.**

| **Advertisement** | **Area- level Income: Odds Ratio and 95% LL and UL Confidence Intervals** | |
| --- | --- | --- |
|  | **Most deprived** | **Least deprived** |
| Unhealthy food and/or drink beverages | 0.90 (0.77 to 1.053) | REF cat |
|  | 0.19 |  |
| Unhealthy food | 0.90 (0.77 to 1.06) | REF cat |
|  | 0.198 |  |
| Sugar-sweetened beverage | 0.96 (0.66 to 1.40) | REF cat |
|  | 0.850 |  |
| Alcohol | 1.02 (0.70 to 1.48) | REF cat |
|  | 0.923 |  |
| E-cigarettes | 0.87 (0.37 to 2.03) | REF cat |
|  | 0.741 |  |
| Gambling | 1.50 (0.47 to 4.80) | REF cat |
|  | 0.492 |  |
| Other | 1.10 (0.96 to 1.27) | REF cat |
|  | 0.180 |  |

**Supplementary Table 4: Coefficients (95% CI) of effect of area-level income deprivation on child exposure by area-level income deprivation, urbanity, sex, and season (Reference categories: Income deprivation = least deprived; Urbanicity = urban; Sex = male; Season = winter)**

| **Advert type** | **Fully Adjusted Model** | **Est.** | **LL 95% CI** | **UL 95% CI** | **P-value** |
| --- | --- | --- | --- | --- | --- |
| **All** | (Intercept) | 0.001 | 0.001 | 0.002 | 0.000 |
| *Pseudo-R2 = 0.064* | **Most vs. least deprived** | **1.454** | **1.087** | **1.945** | **0.012** |
|  | **Urban vs. rural** | **0.443** | **0.244** | **0.805** | **0.008** |
|  | Male vs. female | 0.823 | 0.630 | 1.075 | 0.152 |
|  | Winter vs. summer | 0.845 | 0.644 | 1.108 | 0.222 |
| **Unhealthy Food** | (Intercept) | 0.092 | 0.084 | 0.101 | 0.000 |
| *Pseudo-R2 = 0.149* | **Most vs. least deprived** | **1.175** | **1.055** | **1.309** | **0.003** |
|  | **Urban vs. rural** | **1.285** | **1.042** | **1.583** | **0.019** |
|  | **Male vs. female** | **1.157** | **1.048** | **1.277** | **0.004** |
|  | **Winter vs. summer** | **0.817** | **0.740** | **0.903** | **0.000** |
| **Unhealthy Food & Drink** | (Intercept) | 0.110 | 0.101 | 0.120 | 0.000 |
| *Pseudo-R2 = 0.15* | **Most vs. least deprived** | **1.175** | **1.055** | **1.309** | **0.003** |
|  | **Urban vs. rural** | **1.285** | **1.043** | **1.584** | **0.019** |
|  | **Male vs. female** | **1.157** | **1.048** | **1.277** | **0.004** |
|  | **Winter vs. summer** | **0.817** | **0.739** | **0.903** | **0.000** |
| **Sugar-sweetened Beverages** | (Intercept) | 0.000 | 0.000 | 0.000 | 0.000 |
| *Pseudo-R2 = 0.044* | Most vs. least deprived | 1.330 | 0.603 | 2.938 | 0.480 |
|  | **Urban vs. rural** | **0.067** | **0.005** | **0.899** | **0.041** |
|  | Male vs. female | 1.200 | 0.572 | 2.517 | 0.629 |
|  | Winter vs. summer | 0.682 | 0.322 | 1.444 | 0.317 |
| **Alcohol** | (Intercept) | 0.000 | 0.000 | 0.000 | 0.000 |
| *Pseudo-R2 = 0.044* | Most vs. least deprived | 1.548 | 0.767 | 3.122 | 0.222 |
|  | Urban vs. rural | 0.531 | 0.108 | 2.607 | 0.436 |
|  | Male vs. female | 1.207 | 0.623 | 2.338 | 0.578 |
|  | Winter vs. summer | 1.610 | 0.827 | 3.135 | 0.161 |
| **Other** | (Intercept) | 0.001 | 0.001 | 0.001 | 0.000 |
| *Pseudo-R2 = 0.072* | **Most vs. least deprived** | **1.621** | **1.069** | **2.460** | **0.023** |
|  | **Urban vs. rural** | **0.149** | **0.060** | **0.373** | **0.000** |
|  | Male vs. female | 1.261 | 0.859 | 1.851 | 0.237 |
|  | Winter vs. summer | 0.908 | 0.616 | 1.340 | 0.629 |

Note: Pseudo R2 calculated as 1 - (Residual Deviance / Null Deviance). Note: Statistical significance: *** p < 0.001; ** p < 0.01; * p < 0.05. LL 95% CI – lower level 95% Confidence Interval; UL 95% CI – Upper Level 95% Confidence Interval.
